# Data on distribution, demographic structure and grazing of the dominant mesozooplankton species in the Yenisei estuary and adjacent shelf in early summer

**DOI:** 10.1016/j.dib.2020.105856

**Published:** 2020-06-12

**Authors:** A.V. Drits, A.F. Pasternak, E.G. Arashkevich, S.G. Poyarkov, M.V. Flint

**Affiliations:** Shirshov institute of Oceanology, Russian Academy of Sciences, 36 Nakhimovskii pr., Moscow 117997, Russia

**Keywords:** Kara sea, Yenisei estuary, Zooplankton, Distribution, Herbivorous feeding, Grazing impact

## Abstract

The data article refers to the paper “Distribution and grazing of the dominant mesozooplankton species in the Yenisei estuary and adjacent shelf in early summer (July 2016)” (Drits et al., 2020). The data were collected along quasi-longitudinal transect “Yenisei estuary – Kara Sea shelf” on 24–28 July 2016. Here we present data on the spatial and vertical distribution, demographic structure and gut pigment content of the dominant zooplankton species as well as the grazing impact on autotrophic phytoplankton in the three distinguished zones: freshwater zone, frontal zone of the Yenisei plume and marine shelf zone. The related article (Drits et al., 2020) considers the structure and functioning of zooplankton community in relation to environmental characteristics such as temperature, salinity, phytoplankton abundance, timing of ice retreat. Information presented in this article can be used by marine biologists for studies of structure and functioning of estuarine pelagic communities, ecology of zooplankton in the Siberian seas. Besides the data could provide a baseline for the assessment of the ecological role played by climate change events (e.g., increased precipitation, permafrost thawing, elevated river discharge) on the Arctic ecosystems.

Specifications table

**Table:** 

Subject	Biology
Specific subject area	Zooplankton ecology
Type of data	Tables Figures
How data were acquired	CTD probe (Seabird Electronics SBE-32), Juday closing plankton net (0.1 m^2^ mouth area, 180 µm mesh size), stereomicroscope Leica MZ6, Trilogy Laboratory Fluorometer Turner Designs
Data format	Raw Analyzed
Parameters for data collection	Samples of zooplankton were collected considering data on hydrographic (salinity, temperature) and biological (chlorophyll fluorescence) distribution patterns. Live samples were treated for gut fluorescence analysis immediately after collection. Preserved samples were analyzed for determination of zooplankton abundance and biomass within a few months.
Description of data collection	Mesozooplankton was sampled by vertical tows using a Juday closing plankton net. Zooplankton were identified, staged, measured and counted in the laboratory under a stereomicroscope. Gut pigment content of zooplankters was measured by a fluorometric procedure.
Data source location	Data were collected along a quasi- longitudinal transect “Yenisei estuary – Kara Sea shelf” 24 stations between 75° 55′ and 71° 50′ N.
Data accessibility	With the article
Related research article	A.V. Drits, A.F. Pasternak, E.G. Arashkevich, S.G. Poyarkov, M.V. Flint. Distribution and grazing of the dominant mesozooplankton species in the Yenisei estuary and adjacent shelf in early summer (July 2016). Continental Shelf Research, 2020, 201, 104-133.

## Value of the data

• There is virtually no data on distribution and demographic structure of zooplankton in the Yenisei estuary and adjacent shelf in the early summer (soon after the Yenisei discharge maximum). Our data fill this gap.
• The data are among the few that describe zooplankton feeding and grazing impact on autotrophic phytoplankton in the Yenisei estuary and adjacent Kara sea shelf.
• The data can be used to study the impact of seasonal changes and river run-off in the Arctic shelf seas on the structure and grazing of zooplankton as well as when studying other estuarine pelagic communities.
• The data form a necessary step to assess the effects of climate change in the area of Yenisei influence.

## 1 Data

The data were obtained at 24 sampling stations investigating the distribution and grazing of dominant mesozooplankton species in the Yenisei estuary and adjacent Kara sea shelf in early summer (July 2016). The environmental characteristics of the stations are given in Table 1. The freshwater zone (sts. 5345, 5344, 5343, 5342), frontal zone of the Yenisei plume (sts. 5340, 5339, 5337, 5335, 5333, 5350, 5351) and marine shelf zone outside the plume (5352 and 5353) were distinguished according to salinity and temperature characteristics.

**Table 1:** Stations coordinates, depth (m), surface and bottom salinity (S_sur_, S_bot_), surface and bottom temperature (T_sur_ °C, T_bot_ °C).

Station	Latitude, N	Longitude, E	Depth	S_sur_	S_bot_	T_sur_	T_bot_
5333	74°15,0′	79°10,0′	30	8.3	30.3	11.2	−1.2
5333_2	74°15,1′	79°09,8′	30	7.3	31.0	11.1	−1.4
5334	74°00,4′	79°23,0′	30	6.4	31.1	10.2	−1.4
5335	73°45,7′	79°36,6′	30	5.6	31.9	11.4	−1.4
5335_2	73°45,9′	79°36,1′	30	7.4	31.7	11.5	−1.4
5336	73°33,5′	79°47,4′	38	7.4	32.7	11.3	−1.4
5337	73°18,4′	79°50,9′	32	7.1	31.4	11.8	−1.4
5337_2	73°18,5′	79°50,8′	32	7.7	32.0	11.2	−1.5
5338	73°03,3′	79°55,2′	25	6.8	31.0	11.9	−1.4
5339	72°49,0′	80°00,0′	23	4.9	29.0	12.9	−1.2
5339_2	72°49,0′	79°59,9′	23	5.4	31.1	12.7	−1.4
5340	72°35,9′	80°25,0′	15	5.3	27.6	12.5	−0.3
5340_2	72°35,8′	80°25,4′	15	6.0	28.0	12.6	−0.4
5342	72°12,4′	80°50,3′	12	1.4	26.1	15.6	−0.2
5342_2	72°12,4′	80°50,0	12	0.9	27.9	15.6	−0.2
5343	72°05,6′	81°28,9′	10	1.6	20.9	15.2	6
5344	71°52,0′	82°11,9′	10	0.5	1.3	17.1	16.1
5344_2	71°52,0′	82°12,0′	10	0.6	1.4	16.6	15.9
5345	71°50,4′	082°54,0′	21	0.4	21.4	17.5	6.3
5350	74°52,0′	078°26,9′	22	9.9	29.4	11.6	−1.4
5351	75°12,2′	078°21,1	43	14.2	32.5	11.1	−1.5
5352	75°16,9′	078°18,5′	40	29.6	33.1	6.5	−1.4
5353	75°55,0′	078°34,0′	63	31.4	33.7	3.7	−0.9

Distribution of the biomass of the dominant zooplankton at the sampled stations is presented in Table 2. Cladocera and mysids dominated zooplankton biomass in the freshwater zone. In the Yenisei plume zone, Limnocalanus macrurus dominated zooplankton biomass while *C. glacialis* dominated in the marine shelf zone (Table 2).

**Table 2:** Biomass (mg dry weight m^−3^) of the dominant components of zooplankton along the transect “Yenisei estuary – Kara Sea shelf”.

Station	Species							
	*Calanus glacialis*	*Pseudocalanus* spp*.*	*Limnocalanus macrurus*	*Mysis oculata*	*Bosmina* sp.	*Daphnia* spp.	*Oikopleura vanhoeffeni*	*Others*
5333	12.6	8.5	6.4	3.0	0.0	0.0	1.4	4.7
5333_2	51.9	5.3	7.9	0.0	0.0	0.0	16.0	5.2
5334	13.5	9.6	24.9	0.0	0.0	0.0	25.0	4.6
5335	38.9	8.6	7.7	2.5	0.0	0.0	15.8	6.7
5335_2	5.6	17.5	31.4	0.0	0.0	0.0	15.0	1.6
5336	3.8	10.2	33.1	0.0	0.0	0.0	16.8	3.7
5337	2.6	10.7	17.7	3.2	0.0	0.0	6.8	3.4
5337_2	8.5	18.7	12.7	3.2	0.0	0.0	17.0	6.8
5338	8.6	8.2	1.1	12.6	0.0	0.0	4.5	2.7
5339	1.3	14.7	43.1	0.0	0.0	0.0	0.0	5.3
5339_2	0.0	17.5	9.9	21.8	0.0	0.0	0.0	5.4
5340	0.0	18.5	79.3	0.0	0.0	0.0	0.0	2.2
5340_2	0.0	5.1	38.9	0.0	0.0	0.0	0.0	2.2
5342	0.0	3.6	6.0	0.0	0.7	0.8	0.0	3.4
5342_2	3.4	5.5	11.9	17.0	2.3	2.2	0.0	8.3
5343	0.0	0.0	1.2	0.0	0.8	0.7	0.0	2.7
5344	0.0	0.0	0.0	0.0	10.4	12.6	0.0	5.3
5344_2	0.0	0.0	0.0	1.1	2.8	3.0	0.0	1.3
5345	0.0	0.0	0.0	16.8	3.7	4.1	0.0	2.2
5350	3.2	3.7	4.7	0.0	0.0	0.0	0.0	0.4
5351	17.3	5.0	0.0	0.0	0.0	0.0	6.0	5.0
5352	3.4	5.3	0.0	0.0	0.0	0.0	0.2	3.8
5353	17.6	1.9	0.0	0.0	0.0	0.0	2.0	5.5

Data on the vertical distribution of the dominant zooplankton are presented in Table 3. In the freshwater zone, the dominant zooplankton group, Cladocera, concentrated in the upper water layer. In the Yenisei plume, mesozooplankton avoided the upper freshened layer with *Calanus glacialis* and L. *macrurus* concentrating in the pycnoline layer. *Oikopleura vanhoeffeni* was observed only in the deeper layer, while in the marine shelf zone, they inhabited the upper mixed layer (Table 3). Horizontal and vertical distribution of the dominant zooplankton species was analyzed in relation to temperature, salinity, and Chl *a* concentration using Canonical correspondence analysis (CCA) [1].

**Table 3:** Vertical distribution of abundance (ind m^−3^) of dominant zooplankton species. The layers below, within and above the pycnocline determined according to the CTD profiles were sampled.

Station	Layer, m	Cladocera	*Calanus glacialis*	*Pseudocalanus* spp.	*Limnocalanus macrurus*	*Oikopleura vanhoeffeni*
5333_2	0–5	0	0	102	0	0
	5–10	0	1914	1524	276	6
	10–28	0	52	1344	4	32
5335_2	0–5	0	4	124	0	0
	5–10	0	234	1188	2	2
	10–28	0	13	3431	294	16
5337_2	0–6	0	0	211	0	0
	6–12	0	208	5514	215	2
	12–29	0	24	2055	47	29
5339_2	0–5	0	0	540	12	0
	5–19	0	11	2253	237	0
5340_2	5–0	0	0	58	16	0
	5–12	0	0	930	400	0
5342_2	0–6	7320	0	0	0	0
	6–11	550	0	1670	47	0
5345	0–10	12,980	0	0	0	0
	10–18	350	0	0	0	0
5350	0–5	0	6	452	2	0
	5–10	0	124	1508	82	2
	10–18	0	20	538	8	13
5351	0–10	0	302	320	0	49
	10–38	0	384	1417	0	4
5352	0–10	0	4	7	0	662
	10–37	0	116	1648	0	2
5353	0–20	0	185	1054	0	155
	20–58		720	417	0	4

Data on the demographic structure/size structure of the dominant zooplankters are given in Table 4. Dominance of the younger copepodites of *Calanus glacialis* and *Pseudocalanus* spp, as well as small size *Oikopleura vanhoeffeni* was noted at the marine shelf stations. The population of *Limnocalanus macrurus* consisted of adult specimens with almost equal numbers of females and males.

**Table 4:** Demogaphic structure of the dominant zooplankton species. Abundance (ind m^−3^) of distinguished copepod age stages and larvacean size groups (trunk length, mm) are given.

Station	Calanus glacialis				Pseudocalanus spp.				Limnocalanus macrurus		Oikopleura vanhoeffeni	
	CI	CII	CIII	CIV	CV	CI-CIV	CV	Fem	Fem	Male	<1mm	1–6 mm
5333	1	2	30	62	9	1712	76	15	24	14	1	1
5333_2	5	0	46	280	67	975	112	21	144	132	1	25
5334	1	2	36	52	0	962	151	60	91	56	1	23
5335	1	0	42	218	21	874	152	64	26	19	0	18
5335_2	5	3	14	26	5	1997	458	104	165	129	1	17
5336	0	0	9	7	5	990	234	38	119	76	0	14
5337	1	1	6	8	3	911	268	57	53	54	0	7
5337_2	0	0	6	46	4	1294	595	36	44	27	0	27
5338	1	1	13	45	6	1087	196	21	4	3	0	10
5339	0	0	0	0	0	1462	514	22	134	124	0	0
5339_2	0	0	0	0	0	872	994	20	52	27	0	0
5340	0	0	0	0	0	705	1069	3	244	233	0	0
5340_2	0	0	0	0	0	300	239	0	275	125	0	0
5342	0	0	0	0	0	361	107	0	18	17	0	0
5342_2	0	0	0	0	0	552	187	30	70	68	0	0
5350	5	21	13	3	5	733	41	8	0	0	0	13
5351	119	127	80	35	9	970	110	30	0	0	42	7
5352	29	34	12	4	2	983	152	18	0	0	196	0
5353	223	169	82	43	4	582	37	0	0	0	154	2

**Fig. 1:**
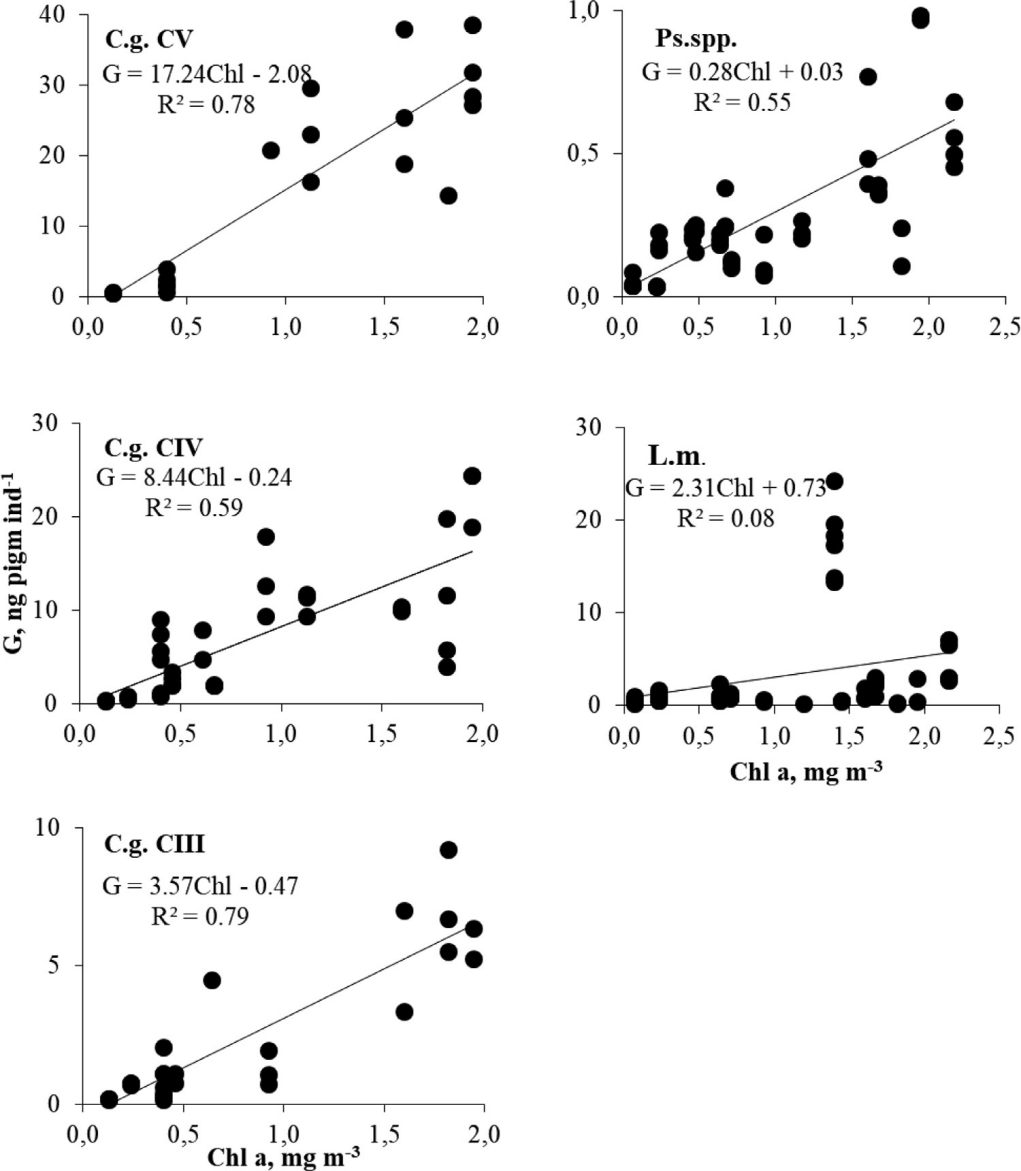
Gut pigment content (G) in dominant zooplankters vs chlorophyll *a* concentration (Chl *a*). L.m. – *Limnocalanus macrurus*, Ps. spp. – *Pseudocalanus* spp. CII-CIV, C. g. – *Calanus glacialis*.

Gut pigment content of copepods depended on chlorophyll concentration in the layer of inhabitance (Fig. 1). A significant correlation between gut pigment content and Chl *a* concentration was found for all copepods but the correlation coefficient was very low for L. *macrurus.*

**Fig. 2:**
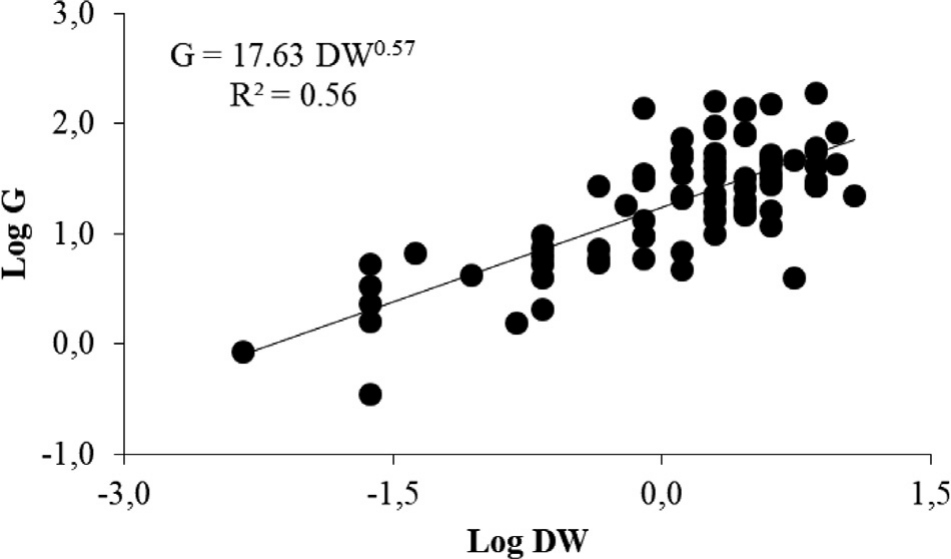
Dependence of *Oikopleura vanhoeffeni* gut pigment content (G, ng pigment ind^−1^) on dry weight (DW, mg ind^−1^).

In *Oikopleura vanhoeffeni,* gut pigment content depended significantly of the weight of the animals (Fig. 2).

Zooplankton grazing on phytoplankton was calculated on the basis of measured gut pigment content of the dominant species and abundance of zooplankters in the sampled water layers (Table 5). The most important grazers were cladocerans (in the freshwater zone) and Calanus glacialis (in the Yenisei plume zone).

**Table 5:** Grazing impact on autotrophic phytoplankton (mg Chl *a* m^−2^ day^−1^) by the populations of the dominant zooplankton species.

Station	Layer, m	Cladocera	*Calanus glacialis*	*Pseudocalanus* spp.	*Limnocalanus macrurus*	*Oikopleura vanhoeffeni*
5333	0–28	0	0.57	0.08	0.004	0
5333_2	0–5	0	0	0.004	0	0
	5–10	0	2.92	0.11	0.02	0
	10–28	0	0.01	0.01	0.1	0.26
5334	0–27	0	0.51	0.09	0.03	0
5335	0–28	0	3.54	0.65	0.04	0
5335_2	0–5	0	0	0.003	0	0
	5–10	0	0.04	0.04	0.008	0.002
	10–28	0	0.006	0.08	0.03	0.22
5336	0–35	0	0.04	0.16	0.08	0
5337	0–29	0	0.03	0.08	0.03	0
5337_2	0–6	0	0	0.007	0	0
	6–12	0	0.05	0.22	0.008	0
	12–29	0	0.014	0.18	0.006	0.18
5338	0–22	0	0.61	0.18	0	0
5339	0–20	0	0	0.92	0.13	0
5339_2	0–5	0	0	0	0	0
	5–19	0	0	0.03	0.012	0
5340	0–12	0	0	0.38	0.42	0
5340_2	0–12	0	0	0.06	0.8	0
5342_2	0–6	0.93	0	0	0.01	0
5344	0–7	3.8	0	0	0	0
5345	0–10	3.03	0	0	0	0
5351	0–10	0	0.03	0.14	0	0.04
	10–38	0	0.05	0.14	0	0.05
5353	0–20	0	0.017	0.03	0	0.004
	20–58	0	0.42	0.015	0	0.02

## 2 Experimental design, materials and methods

### 2.1 Collection and processing of samples

**Fig. 3:**
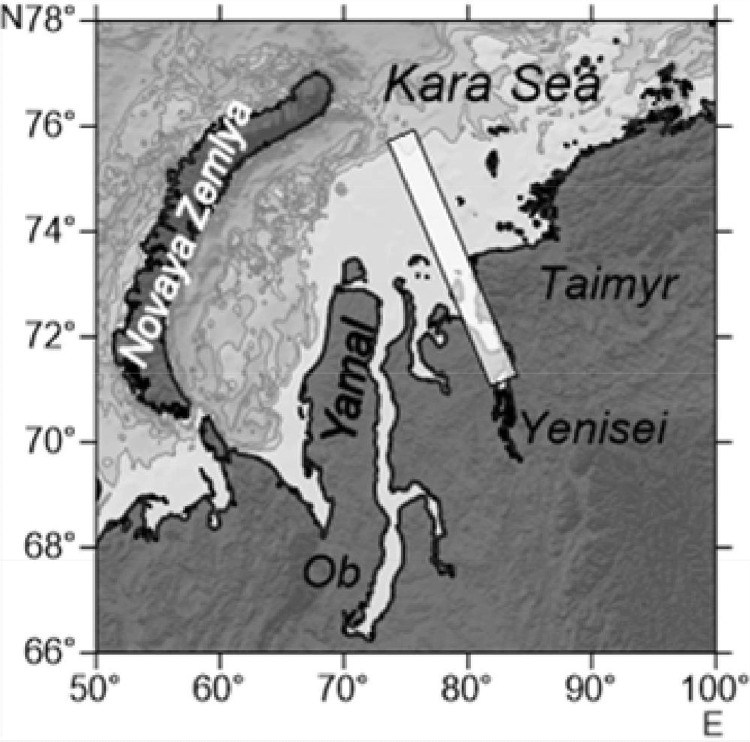
Map of the study area: location of the transect is indicated with a bar.

The material was collected during cruise # 66 of the RV “Akademik Mstislav Keldysh” to the Kara Sea in July 2016. A quasi-longitudinaltransect was sampled in the Yenisei estuary and adjacent shelf (Fig. 3). At first, the transect was sampled from 74°15′ N to 71° 50′ N on 24–25 July. Based on the obtained salinity and temperature data, several stations were selected and repeated along the reversed transect from 71° 50′ N to 76° N on 26–28 July. The coordinates of the sampling stations are in Tables 1. Data on temperature and salinity were obtained with a CTD probe (Seabird Electronics SBE-32). Mesozooplankton was sampled using a Juday closing net (0.1 m^2^ mouth area, 180 µm mesh size). Two layers were sampled at most of the stations along the SN transect, below and above the pycnocline, which was determined according to the CTD profiles. Additionally, we sampled the pycnocline layer at four of the reversed transect stations. For determination of zooplankton abundance and biomass, samples were immediately preserved in 4% borax-buffered formalin. Zooplankton were identified, staged, measured and counted in the laboratory under a stereomicroscope. For determination of zooplankton abundance and biomass, samples were immediately preserved in 4% borax-buffered formalin. Zooplankton were identified, staged, measured and counted in the laboratory under a stereomicroscope. Not numerous large specimens (the older stages of *Calanus* spp., *Limnocalanus macrurus, Oikopleura vanhoeffeni* and Chaetognatha) were counted in the whole samples, while more numerous forms were counted in subsamples so as not less than 50 specimens were recorded. Most taxonomic groups including Copepoda, Cladocera, Pteropoda, Chaetognatha, Larvacea, Mysidacaea were identified to the species/genus level. Prosome length was used to distinguish the closely related copepod species *Calanus finmarchicus* and *C. glacialis* according to [2]. For *Calanus* and *Limnocalanus*, all copepodite stages were distinguished. For *Pseudocalanus* spp. copepodites CI to CIV were pooled. The wet weight (WW) of each species was calculated using nomograms [3]. These tables (www.twirpx.com/file/1588162/) allow the calculation of biovolume/wet weight of aquatic organisms based on their body shape and length. Dry weight (DW) of crustacean plankton was estimated as 0.16 WW [4], DW of chaetognaths was calculated according to [5], larvaceans – to [6].

### 2.2 Feeding of zooplankton

Feeding rates of the dominant species (*Calanus glacialis, Limnocalanus macrurus, Pseudocalanus* spp., *Bosmina* sp*., Daphnia* spp*., Oikopleura vanhoeffeni*) were assessed with the gut fluorescence method [7]. To measure gut pigment content, 1 to 40 animals per replicate, depending on size/stage, were picked with forceps and placed in test tubes with 3 ml of 90% acetone. Two to five replicates for each species/stage were analyzed. Pigments were extracted for 24 h at 5 °C in the dark. Chl *a* and phaeopigment were measured by a standard fluorometric procedure [8] with a Trilogy Laboratory Fluorometer (Turner Designs).

### 2.3 Zooplankton grazing impact

Grazing impact of each of the dominant species (mg Chl *a* m^−2^ day^−1^) was estimated using the mean individual daily ingestion rates (ng Chl *a* ind^−1^ d^−1^) and the abundance of the given species (ind m^−2^).

## Declaration of Competing Interest

None.

## Appendix Supplementary materials

Image, application 1
